# Multisystem inflammatory syndrome in children is a SARS-CoV-2 triggered Kawasaki disease

**DOI:** 10.1038/s41390-025-04499-8

**Published:** 2025-10-27

**Authors:** Greta Mastrangelo, Paul Tsoukas, Ellen Go, Hua Lu, Arthur Hoi Hin Cheng, Amy Xu, Rae S. M. Yeung

**Affiliations:** 1 Division of Rheumatology, Department of Paediatrics, The Hospital for Sick Children, University of Toronto, Toronto, ON Canada; 2 Cell and Systems Biology Program, The Hospital for Sick Children Research Institute, Toronto, ON Canada; 3 Division of Pediatric Rheumatology, Indiana University Health Medical Center, Atlanta, GA USA; 4 Department of Immunology and Institute of Medical Science, University of Toronto, Toronto, ON Canada; 5 The Hospital for Sick Children, University of Toronto, Toronto, ON Canada; 6 Division of Cardiology, Department of Paediatrics, The Hospital for Sick Children, University of Toronto, Toronto, ON Canada; 7 Division of Rheumatology and Dermatology, Department of Paediatrics, Children’s Hospital of Eastern Ontario, Ottawa, ON Canada; 8 Department of Paediatrics, The Hospital for Sick Children, University of Toronto, Toronto, ON Canada; 9 Division of Rheumatology, Department of Paediatrics, Centre Hospitalier Universitaire Sainte-Justine, Montreal, QC Canada; 10 The Rosalind & Morris Goodman Family Pediatric Formulations Centre of the CHU Sainte-Justine, Montreal, QC Canada; 11 Division of Rheumatology, St. Michael’s Hospital, Toronto, Canada; 12 Division of Emergency Medicine, Department of Paediatrics, The Hospital for Sick Children, University of Toronto, Toronto, ON Canada; 13 Division of Infectious Disease, Department of Paediatrics, The Hospital for Sick Children, University of Toronto, Toronto, ON Canada; 14 Present Address: Division of Pediatric Rheumatology, Indiana University Health Medical Center, Atlanta, GA USA; 15 Present Address: Division of Rheumatology, Department of Paediatrics, The Hospital for Sick Children, University of Toronto, Toronto, ON Canada

## Abstract

**Background:**

Multisystem Inflammatory Syndrome in Children (MIS-C) encompasses a spectrum of phenotypes: shock, Kawasaki disease (KD), and fever with hyperinflammation. Whether MIS-C is a new syndrome or SARS-CoV-2-triggered KD remains debated. To explore this, we investigated the relationship between clinical phenotypes and viral variants, and the contribution of pre-pandemic KD incidence to MIS-C reporting.

**Methods:**

Single center, prospective, observational study of 384 patients with MIS-C, from March 2020 to September 2023. Clinical and laboratory features, complications, and outcomes were evaluated across the MIS-C waves.

**Results:**

Three clinical phenotypes were identified: shock, KD, and fever with hyperinflammation. KD was most common across all variants, particularly during Omicron, while shock predominated in Delta cohort. Stratifying by phenotype outperformed the WHO MIS-C and RCPCH PIMS definitions in distinguishing subgroups. Countries with low pre-pandemic KD incidence identified MIS-C as a new syndrome, while countries with high KD incidence did not.

**Conclusions:**

MIS-C phenotypes vary accordingly to SARS-CoV-2 variants, with KD being most common. Stratification by clinical phenotypes out-performed MIS-C case definitions for patient identification, highlighting the value of clinical features in managing infection-triggered hyperinflammation. These findings, coupled with the inverse relationship between pre-pandemic KD incidence and MIS-C reporting, support the hypothesis that MIS-C is SARS-CoV-2-triggered KD.

**Impact:**

- KD and MIS-C are not separate entities but different ends of the immune response spectrum.
- Among the hyperinflammation spectrum, each viral variant induces a distinct MIS-C phenotype, with the Omicron wave resembling KD.
- Clinical phenotype stratification outperformed MIS-C definitions in identifying patient subgroups, confirming the value of clinical features in managing infection-triggered hyperinflammation.
- The inverse relationship between pre-pandemic KD incidence and MIS-C reporting supports MIS-C being a SARS-CoV-2–triggered KD, underscoring critical equity and diversity considerations.
- A pathogen-agnostic approach to post-infectious hyperinflammation would recognize the full spectrum of phenotypes and complications and avoid confusion due to new naming conventions.

## Introduction

Multisystem inflammatory syndrome in children (MIS-C) is a hyperinflammatory disorder characterized by fever and multiorgan dysfunction associated with SARS-CoV-2. Differing case definitions have been proposed including: Pediatric inflammatory multisystem syndrome temporally associated with COVID-19 (PIMS-TS),^1,2^ the term coined by the Royal College of Paediatric and Child Health (RCPCH) in the United Kingdom and Canadian Paediatric Surveillance Program (CPSP), and MIS-C,^3,4^ the name used by the World Health Organization (WHO) and Centers for Disease Control (CDC). Affected children present with features similar to those of Kawasaki disease (KD) and toxic shock syndrome.^5–7^ Validated diagnostic criteria do not exist, with some professional organizations using the same nomenclature but different criteria to define the syndrome. As a result, researchers have employed varying case definitions of MIS-C resulting in patient populations that are not necessarily comparable across studies.^7^ MIS-C encompasses a wide disease spectrum of clinical phenotypes with cardiogenic shock and macrophage activation syndrome (MAS) at the severe end and a self-limited febrile illness at the mild end.^8–10^ Three clinical patterns are recognized at the bedside: cardiogenic shock, KD, and fever with hyperinflammation.^2,8^ The SARS-CoV-2 virus has evolved throughout the pandemic resulting in variants that differ in acute illness, transmissibility, outcomes, vaccine effectiveness,^11^ and potentially in MIS-C phenotypes.

In this study, we characterized a multi-ethnic cohort of children with MIS-C across the various waves of SARS-CoV-2 infection (Ancestral/pre-Delta, Delta, and Omicron MIS-C cohort). We evaluated the impact of viral variants on clinical phenotypes, the effect of stratification by clinical phenotype in capturing homogeneous patients’ groups compared to the WHO MIS-C/RCPCH PIMS case definitions, and the impact of pre-pandemic KD incidence on the geographic variations in MIS-C reporting.

## Methods

### Study design and participants

Single-center, prospective, observational study of 384 consecutive, hospitalized patients treated for MIS-C, at a tertiary care pediatric center in Canada from March 7, 2020 to September 8, 2023. All suspected cases, less than 18 years of age, temporally linked to SARS-CoV-2, with or without microbiological or serological confirmation, who were treated as MIS-C,^12^ were reported. All patients were reviewed in real-time and evaluated by a multidisciplinary MIS-C working group to determine diagnosis and management approach, and case assignments according to the study definitions (see “Supplementary Methods”). The definition of MIS-C included children who (i) met the most permissive case definition according to international MIS-C and PIMS definitions^1–4^ and (ii) were adjudicated in real-time by the physician at the bedside confirmed by the SickKids multidisciplinary MIS-C working group as having MIS-C and (iii) were hospitalized and treated for the condition. MIS-C phenotypes definitions were based on published reports,^2,8^ and include cardiogenic shock, KD, and fever with hyperinflammation.

All patients followed a standardized management pathway, with prospective, protocolized collection of core clinical data including demographics, clinical features, laboratory measurements, measures of disease severity (length of hospital stay, intensive care admission, respiratory and inotropic support, fluid resuscitation), complications including shock and MAS, and cardiac outcomes such as the presence of coronary artery aneurysms, and electrocardiogram abnormalities.

Given the lack of individual-level variant confirmation, the association with SARS-CoV-2 variants and MIS-C was assigned based on local epidemiology and sequencing data, representing the predominant variant across three time periods: (i) March 7, 2020 through April 20, 2021, for the Ancestral, Alpha, Beta, and Gamma variants; (ii) April 21, 2021 through December 13, 2021, for the Delta variant; and (iii) December 14, 2021 through September 8, 2023, for the Omicron variants. The start date of each corresponding MIS-C wave, for study purposes, was set at two weeks from the peak of acute COVID-19 from that respective variant in the community corresponding to the known time lag of two-six weeks for developing MIS-C after the acute infection.^12^ The following MIS-C surges were identified: Ancestral/pre-Delta (Ancestral/Alpha/Beta/Gamma), Delta, and Omicron MIS-C waves.

This study was approved by The Hospital for Sick Children Research Ethics Board, and the requirement for individual consent was waived.

### MIS-C incidence

The number of COVID-19 cases reported to Toronto Public Health between 2020-01-23 and 2022-11-15 published on City of Toronto website^13^ were used to calculate a 7-day moving average of COVID-19 case count. These case counts were then used as the denominator when calculating the MIS-C incidence rates. The number of MIS-C cases seen at the Hospital of Sick Children was used as numerator.

### Ethnicity

To compare the population’s make-up (ethnicity) during and pre the various SARS-CoV-2 variants waves we used pie charts to visualize the various ethnicities including comparisons to the general population based in the city of Toronto^14^ and those with acute COVID-19 at the beginning of the pandemic.^13^

### Comparison between case definitions

To compare the ability to capture homogenous groups of patients between clinical phenotypes and the WHO MIS-C/RCPCH PIMS case definitions, we used the empirical cumulative distribution functions (eCDFs) of *p*-values that were considered a measure of differentiation by examining the distance between distributions. They were plotted to depict the strength of clinical phenotype stratification and various case definitions. For each classification method, we converted the k univariate *p*-values into an empirical cumulative distribution function (eCDF). Let ${p}_{\left(1\right)},\ldots ,{p}_{\left(k\right)}$ be the sorted *p*-values. For any threshold t in [0,1], the eCDF is

$$\hat{F}\left(t\right)=\frac{1}{k}{\sum }_{i=1}^{k}{{{\bf{1}}}}\{{p}_{\left(i\right)}\le t\}$$

that is, the proportion of *p*-values no larger than t. Plotting $\hat{F}\left(t\right)$ against t yields a step function whose height at each t reflects the fraction of clinical variables achieving that significance level. The approach follows the eCDF of *p*-values methodology previously used to benchmark phenotype-stratification pipelines in imaging.^15,16^

### Regional differences in incidence of KD, SARS-CoV-2, and MIS-C

To examine how baseline KD incidence might influence recognition of MIS-C, we modeled annual KD incidence over the past five decades from reported data from Japan, Taiwan, Ontario (Canada), the United States (USA), the United Kingdom (UK), and Italy using a Gaussian generalized linear model with the log-link function.^17–25^ For each country, we generated a 95% prediction interval (PI) around the model fit, an uncertainty measurement of KD case ranges predicted in each year. Using the most recent decade of data, we then calculated a “noise factor” by dividing the upper limit of the 95% PI over their corresponding expected values for each year, and report its maximum as an estimate of natural KD case fluctuation (noise estimate).

To assess the contribution of the incidence of SARS-CoV-2 infection and KD on reporting of MIS-C, we calculated a simple ratio for each country by dividing the peak COVID-19 incidence rate (cases per 1,000,000 population)^26^ during the first-wave of COVID-19, by the pre-pandemic KD incidence rate (cases per 100,000 children <5 years).^17–25^ This number is an estimate of the number of COVID-19 cases per ten KD cases in each country. Furthermore, we calculated the ratio between the MIS-C incidence per 100,000 SARS‑CoV‑2 infections/ year in Japan, Taiwan, Ontario (Canada), USA, UK, and Italy^27–34^ and the pre-pandemic KD incidence rate based on the most recent reported data available prior to 2020 for each country.^17–25^

### Descriptive statistics

Descriptive statistics were used for variables including clinical and laboratory features, severity measures, complications, and outcomes. Continuous variables were presented as median with interquartile range, and categorical variables were presented as frequency. Wilcoxon rank-sum test and Kruskal-Wallis tests were performed to compare continuous measurements as appropriate. For categorical variable comparisons, Fisher exact test or chi-square test of independence without continuity correction was used as appropriate. Analyses were conducted using R, version 4.0.2 (R Foundation for Statistical Computing, Vienna, Austria). Various approaches to the analyses were performed, including those for the entire group as well as subgroup analysis restricted only to patients with confirmed SARS-CoV-2 infection and those who strictly met the WHO MIS-C case definition.

## Results

### Epidemiology varies across SARS-CoV-2 waves

**Fig. 1:**
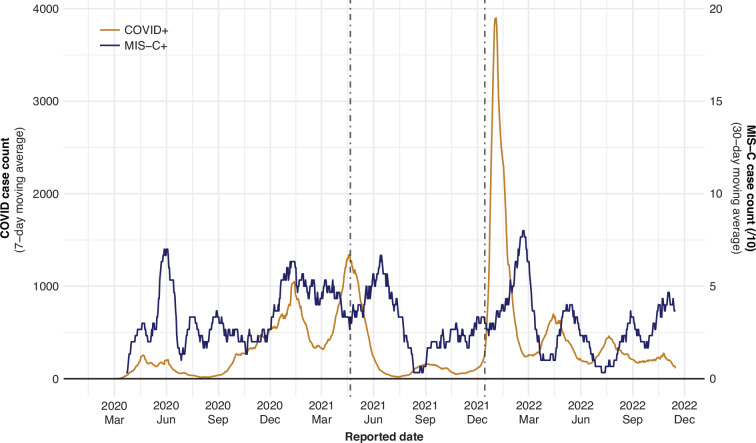
Incidence of acute COVID-19 and MIS-C. MIS-C cases (brown line) admitted to The Hospital for Sick Children and COVID-19 incidence (blue line) in the city of Toronto across the various SARS-CoV-2 waves. The MIS-C and COVID-19 incidence are plotted as seven and 30 moving average, respectively.

Three hundred and eighty-four children with a working diagnosis of MIS-C admitted to a tertiary care children’s hospital between March 7, 2020, and September 8, 2023, were included in this study. The median age was 5.0 years (IQR, 2.0–9.0) and 58% were male, 10% of patients were vaccinated with at least one dose at presentation (Supplementary Table S1). One hundred forty patients were included in the MIS-C cohort associated with the Ancestral/pre-Delta wave, 71 associated with the Delta variant, and 173 with the Omicron variant (Supplementary Table S2). The pattern of disease onset showed MIS-C cases following the surge of COVID-19 in the community by two-six weeks (Fig. 1). Interestingly, the incidence rate of MIS-C differed depending on the viral variant, with 1.2, 1.1, and 0.5 MIS-C cases per 1000 SARS-CoV-2 cases, in the Ancestral/pre-Delta, Delta, and Omicron waves, respectively.

One hundred twenty-three children (32%) of self-reported Asian ethnicity represented the largest subgroup, with 72 from East-Asian and 51 from South-Asian backgrounds. We compared ethnicity data from our study to local population statistics^14^ and COVID-19 infection rates^13^ (Supplementary Fig. S1A). Our local data showed that racial minority groups (South Asian, Black, Latin American, and Arab communities) had higher rates of SARS-CoV-2 infection than expected based on the general population early in the pandemic (Supplementary Fig. S1B). These proportions were also reflected in our Ancestral/pre-Delta and Delta MIS-C cohorts (Supplementary Fig. S1D, 1E). As the pandemic evolved, more East-Asian individuals were affected, which was shown in the Omicron MIS-C cohort (Supplementary Fig. S1F), mirroring the distribution of ethnicities seen in pre-pandemic KD (Supplementary Fig. S1C).

### MIS-C phenotypes vary between SARS-CoV-2 variants

When the MIS-C clinical phenotypes (cardiogenic shock, KD, and fever with hyperinflammation) were compared across the various MIS-C waves, the three cohorts showed a significant difference in their distribution (*p* ≤ 0.001). The KD phenotype was the most common, observed in 63%, 52% and 80% of children during the Ancestral/pre-Delta, Delta, and Omicron MIS-C waves, respectively. Fever with hyperinflammation was observed most (21%) in the Ancestral/pre-Delta cohort; shock phenotype was the highest (35%) in the Delta cohort; and the KD phenotype was highest (80%) in the Omicron cohort (Table 1). Similar results were seen when the cohort was restricted to either those who fulfilled the WHO MIS-C case definition (Supplementary Table S3A), or to only confirmed SARS-CoV-2 exposed children (Supplementary Table S3B). In the Omicron cohort, each individual KD feature was more common at presentation and was thus associated with more children presenting with complete KD (58%) compared to the other cohorts (Table 2). The proportion of children developing coronary artery lesions and electrocardiogram abnormalities was similar in all groups. A higher proportion of patients in the Delta MIS-C cohort presented with shock (35%) and MAS (20%), which was reflected in those requiring intensive care admission (31%), and in laboratory investigations with lower lymphocytes and albumin, higher ferritin, D-Dimer, and N-terminal pro-brain natriuretic peptide. Omicron patients had lower sodium, and higher erythrocyte sedimentation rate, typically associated with classic KD (Supplementary Table S2). These findings were also observed when only those who fulfilled the WHO MIS-C case definition or only SARS-CoV-2 positive children were considered (data not shown).

**Table 1:** MIS-C clinical phenotypes across MIS-C waves

MIS-C phenotypes	Ancestral/pre-delta, (*n* = 140^a^)	Delta, (*n* = 71^a^)	Omicron, (*n* = 173^a^)
Fever with hyperinflammation	30 (21%)	9 (13%)	5 (3%)
KD	88 (63%)	37 (52%)	139 (80%)
Shock	22 (16%)	25 (35%)	29 (17%)

*KD* Kawasaki disease.

^a^No. (%).

**Table 2:** KD features across MIS-C waves

	Ancestral/pre-delta, (*n* = 140^a^)	Delta, (*n* = 71^a^)	Omicron, (*n* = 173^a^)	*p*-value^b^
Rash	79 (56%)	40 (56%)	135 (78%)	<0.001*
Peripheral extremity changes	49 (35%)	29 (41%)	112 (65%)	<0.001*
Cervical lymphadenopathy	33 (24%)	23 (32%)	80 (46%)	<0.001*
Oromucosal changes	70 (50%)	39 (55%)	138 (80%)	<0.001*
Bilateral non-exudative conjunctivitis	81 (58%)	55 (77%)	142 (82%)	<0.001*
Number of KD features^c^	2.0 (1.0, 3.0)	3.0 (1.0, 4.0)	4.0 (3.0, 4.0)	<0.001*
Complete KD	29 (21%)	21 (30%)	101 (58%)	<0.001*

*KD* Kawasaki disease.

^a^Median (IQR); No. (%); *n*/*N* (%)

^b^Pearson’s Chi-squared test

^c^KD features included: fever ≥5 days of fever, rash, extremity changes, oral mucosal changes, cervical lymphadenopathy (≥1.5 cm diameter), and bilateral conjunctivitis.

*p value < 0.05.

### Stratification by clinical phenotypes captures more homogeneous patient groups

When patients were stratified by clinical phenotypes,^2,8^ cardiogenic shock represented 20%, KD 69%, and fever with hyperinflammation 12% of patients. Patients with shock were older, with higher incidence of gastrointestinal, neurological, renal, and cardiac involvement, together with MAS, and required more intensive care management (Supplementary Table S4). The identical observations were seen when only those satisfying the WHO MIS-C case definition or with confirmed SARS-CoV-2 exposure were included (data not shown).

**Fig. 2:**
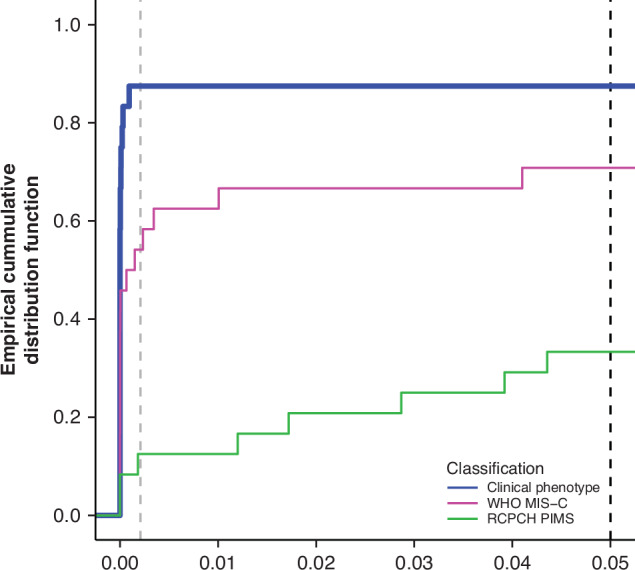
Ability of classifications to distinguish patients on clinical features. For each classification, the eCDF (*y*-axis) for p-values (*x*-axis). Any given point (*x*, *y*) denotes the proportion (*y*) of tested clinical features with a *p*-value ≤ *x*. The vertical dashed black line shows a significance level of 0.05 and the dashed gray line shows the Bonferroni-adjusted significance level of 0.05/24 = 0.0021.

Clinical phenotypes observed at the bedside distinguished between unique groups of patients better than the WHO MIS-C or RCPCH PIMS case definitions, as shown by eCDF analysis of *p*-values (Fig. 2). Across all *p*-value thresholds, more variables significantly differentiated patient subgroups when using clinical phenotypes compared to MIS-C or PIMS case definitions. For instance, at *p* < 0.05, 21/24 (87.5%) variables showed significant differences using the clinical phenotypes classification, compared to 17/24 (70.8%) for WHO MIS-C and even less, 7/24 (29.2%), for RCPCH PIMS. Even with family-wise error rate adjustments, 20/24 (83%) clinical features continue to be distributed differently between clinical phenotypes, with 8 of these 20 features (40%) being similar in the WHO classification, and 17/20 (85%) in the RCPCH definition.

### Comparison of KD incidence between countries

**Fig. 3:**
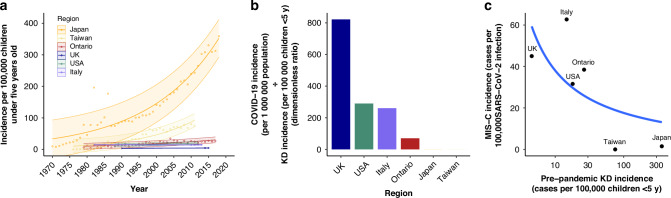
Association pre−pandemic KD rate with frequency of MIS−C cases. **a** Linear model-for each country (colors: top−left legend), gaussian generalized linear models and their 95% prediction intervals (PI) were fit to the KD incidence (*y*−axis) over time (*x*−axis). **b** Cumulative SARS−CoV−2 cases divided by the pre−pandemic KD incidence (the values represent the number of COVID−19 cases for every 10 KD cases expected in the same population) for the countries shown. **c** MIS-C cases divided by pre-pandemic KD incidence. The values represent the number of MIS-C cases for every KD case in the countries shown.

KD incidence rates vary significantly across the globe, with up to a 40-fold difference between East Asia^17–20^ and Europe.^21,22^ We examined the contribution of pre-pandemic KD incidence on MIS-C reporting worldwide. Using data from Japan, Taiwan, Ontario (Canada), the USA, UK, and Italy from the pre-pandemic years, we estimated expected KD case numbers and their normal yearly variation. We then calculated the variation in KD cases i.e., the potential increase and decrease in cases in each country by comparing the expected values to the upper limit of this variation (noise estimate). The maximum noise estimate for Japan is 1.3, followed by Taiwan at 1.4, Ontario at 1.5, USA at 1.3, Italy at 1.1, and the UK at 1.1, with the corresponding change in incidence of 60.2, 6.6, 4.6, 1.2, and 1.1 cases per 100,000 children, respectively (Fig. 3a). This means that to recognize an unusually high case-load of KD out of keeping with previous trends, Japan needs to see 1.3 times more KD cases than they normally see, translating to an increase in incidence of 60 cases per 100,000. Whereas, in a low KD incidence country like the UK, they only need to observe 1.1 times more KD cases than previously seen, corresponding to an incidence’s increase of only 0.5 cases per 100,000 to recognize the signal as unusually high (signal to noise estimate). Thus, a small increase in the absolute number of KD cases above baseline in the UK would be recognized as unusual, whereas in Japan, that increase in number of cases is 120-fold greater when compared to the UK. Thus, to recognize a signal which is out of the ordinary, Japan needs to see 120 times more KD cases compared to the UK.

To understand how COVID-19 and KD rates may have affected MIS-C reporting, we calculated the ratio of peak COVID-19 cases during the first COVID-19 wave to pre-pandemic KD cases in each country. This gave an estimate of how many COVID-19 infections occurred for every 10 KD cases. The UK has the highest ratio at 821.5, followed by USA at 289.9, Italy at 260.7, Ontario at 70.3, Japan at 0.4, and Taiwan at 0.2 (Fig. 3b). When comparing the ratio of MIS-C reporting to pre-pandemic KD incidence across countries, Japan and Taiwan had the lowest ratios (0 and 0.004, respectively), while Italy and the UK had the highest (4.27 and 9.89, respectively). Ontario’s ratio was 1.46, and the USA’s was 1.76 (Fig. 3c). These data echo the geography and reporting of MIS-C cases and point to an inverse relationship between MIS-C reporting and pre-pandemic KD incidence amplified by the local incidence of SARS-CoV-2.

## Discussion

Our results suggest that KD and MIS-C are not entirely separate conditions, but rather found at different points along a shared spectrum of immune response. From a demographic perspective, as the pandemic progressed and spread broader through the population, we observed an increasing number of cases among individuals of Asian descent, similar to the demographic pattern seen prior to the pandemic in children with KD. From a clinical perspective, the different MIS-C waves represent just the visible tip of the iceberg of a broader spectrum of infection-triggered hyperinflammation, with the most recent SARS-CoV-2 viral variant resulting in more classic KD phenotype, thus contributing to the misconception that MIS-C is no longer seen. To support this hypothesis, we conducted two key comparisons. First, we analyzed how well clinical phenotypes identified at the bedside could identify meaningful, homogeneous patients’ groups compared to the WHO and RCPS MIS-C case definitions. We found that bedside clinical assessment outperformed standardized case definitions, which tend to capture only the most severe end of the MIS-C spectrum. Second, we compared MIS-C reporting in countries with different pre-pandemic KD incidence. Our results suggested that one potential contributor to MIS-C being labeled as a distinct condition was that the severe inflammatory presentation in Western countries was not only less common, leading to more noise and reporting of an unfamiliar signal but coupled with a higher incidence of COVID-19 at the beginning of the pandemic. In contrast, in East Asian countries, where KD is much more common, the noise estimate was different and the extreme phenotypes of KD were already known pre-pandemic, thus with such cases were classified simply as KD, and not a new syndrome named MIS-C.

At the beginning of the pandemic, MIS-C was reported in racialized children, echoing the skewed incidence of acute COVID-19 in these populations. Interestingly, on further examination in our cohort, regardless of MIS-C wave, Asian children remained overrepresented, similar to pre-pandemic KD patterns. This was most evident in the Omicron MIS-C cohort, where Asians comprised 51%, reflecting the predominant KD phenotype in this cohort. This aligns with the known genetic predisposition to KD, with Asians being more susceptible than Caucasians, as well as with recent publications focused on MIS-C ethnicity distribution.^35,36^ Our results support that the viral triggers play an important role in determining the clinical phenotype, while genetics likely contributes to disease susceptibility. MIS-C appears to be a SARS-CoV-2-triggered inflammatory syndrome, with each viral variant leading to a different clinical phenotype differing in intensity of inflammation.^37,38^ KD remained the predominant phenotype throughout all the MIS-C waves, representing 80% of children during the Omicron wave. Early clinical studies suggested that on clinical features alone, KD and MIS-C might belong to a common spectrum of pathogen-triggered hyperinflammatory states.^8–10^ More recently, it was shown that KD and MIS-C have a share host immune response as measured by gene expression signatures, pointing to a shared disease spectrum.^38^ Our findings, on a clinical level, support the hypothesis that the early MIS-C descriptions represent an extreme phenotype of KD triggered by a new viral entity. This is supported by the studies that showed that the extreme presentations as MIS-C have distinct molecular phenotypes at the protein, transcriptomic, and cell-free RNA levels compared to the less severe phenotypes in pre-pandemic KD, a logical conclusion.^39^ Indeed, the varying MIS-C phenotypes seen with SARS-CoV-2 variants align with historical descriptions of varying KD phenotypes with different infectious triggers.^40^ Now SARS–CoV-2 can be added to the list of infectious agents associated with KD, with KD and MIS-C as names describing the different ends of a spectrum of the host immune response.

These data highlight the tension between rapid recognition and case surveillance early in the pandemic, and the need for better understanding of symptoms and more comprehensive case definitions. The MIS-C case definitions were crucial for surveillance during the early stages of the health emergency, yet they aren’t ideal diagnostic instruments, as they only capture the most severe presentations. This bias limits the ability to capture of the whole spectrum of SARS-CoV-2-triggered hyperinflammation, and has continued to skew our understanding of MIS-C. An alternative approach is to stratify post-infectious hyperinflammation syndrome into subgroups based on the clinical phenotype recognized at the bedside, in a manner agnostic to the infectious trigger.

The value of relying on clinical phenotypes at the bedside was confirmed when we evaluated their ability at capturing homogeneous patients’ subgroups. Stratifying patients into well-known clinical phenotypes of shock, KD, or fever with hyperinflammation, based on clinical judgment, performs better than any of the case definitions at distinguishing between patients’ groups. This is true no matter how patient groups are divided based on case definitions and/or SARS-CoV-2 exposure. This highlights that SARS-CoV-2 is just one of many possible infections that can trigger hyperinflammation in children, and it shows the value of using known clinical phenotypes and bedside judgment to help recognition and management of children with infection-triggered hyperinflammation.

Our hypothesis that MIS-C is SARS-CoV-2-triggered KD is also supported by the observations of MIS-C reporting from various countries. Non-Asian countries were the first to report MIS-C because the signal-to-noise ratio in these low KD-incidence countries was very high compared to Asian countries during the beginning of the COVID-19 pandemic. In contrast, the KD cases triggered by SARS-CoV-2 in Japan and Taiwan were still within their normal variation, given the higher baseline KD incidence. In high case-load countries, the higher endemic KD rates may also contribute to the recognition of the more extreme presentations of shock and MAS as part of the spectrum of KD before the pandemic, and not a new disease entity, as proposed by low KD case-load countries. Indeed, KD shock and KD MAS phenotypes are well-known entities, accounting approximately 1.4–7% and 2–5% of all KD cases, respectively.^41,42^ Findings from KD shock and KD MAS are similar to early reports of MIS-C^5–7^ and represent the most severe end of the KD/MIS-C spectrum. As a result, current case definitions tend to capture only these severe cases, missing the milder forms that look more like typical KD.

Our data support the notion that MIS-C is along the KD spectrum, consistent with the knowledge that KD is heterogeneous with infectious agent outbreak-dependent phenotypes.^43^ The inverse relationship between pre-pandemic KD incidence and MIS-C reporting highlights also a significant difference between Eastern and Western nations, underscoring critical equity and diversity considerations. Recognizing these differences necessitates addressing variations in clinical experience, healthcare infrastructure, and regional disease incidence to promote equitable and inclusive diagnostic and treatment practices on a global scale.

Our study has limitations. Not all presumed MIS-C patients had SARS-CoV-2 serology testing performed, as serologic testing was not available until August 2020 and government-funded testing remained restricted with routine testing ending in 2024. We were unable to confirm the viral strain in each patient, thus, we assigned patients to variant groups based on the dominant strain at the time, without individual-level confirmation. This may lead to a risk of misclassification bias if some patients were infected with a different variant. The lower incidence of MIS-C during the Omicron wave may result from fewer tertiary-center transfers due to heightened community-hospital confidence and disease awareness. We recognize that the addition of molecular-level data would have enabled more precise subtyping of hyperinflammatory clinical syndromes and offered deeper insight into their underlying pathophysiology. However, this was beyond the scope of this study.

## Conclusion

KD and MIS-C are not separate entities but are names describing the different ends of the spectrum of the post-infectious host immune response. MIS-C is a SARS-CoV-2-triggered KD and each viral variant leads to a different clinical phenotype. While case definitions performed well for surveillance, they introduced new challenges by renaming existing diseases, and overlooked less extreme phenotypes. Our data show that stratifying post-infection hyperinflammation syndrome by clinical phenotype recognized at the bedside, in a pathogen-agnostic approach identifies homogeneous patients better than the WHO MIS-C/RCPCH PIMS case definitions. Such an approach would recognize the full spectrum of phenotypes and complications and avoid confusion due to new naming conventions. In high KD-incidence countries, the higher endemic KD rates contributed to the recognition of the extreme presentations as part of the spectrum of KD, and not a new disease entity, as proposed by low KD-incidence countries, explaining the inverse relationship between pre-pandemic KD incidence and MIS-C reporting, highlighting critical equity and diversity considerations.

## Supplementary information

Supplementary materials

## Data Availability

The de-identified datasets analyzed during the current study are available from the corresponding author on a written reasonable request and after adjudication by the study data access committee.
