# Supplementary material for: Multisystem inflammatory syndrome in children is a SARS-CoV-2 triggered Kawasaki disease
Source: Pediatr Res. 2025 Oct 27;99(6):2267–73. doi: 10.1038/s41390-025-04499-8 (PMC13433241; doi:10.1038/s41390-025-04499-8)
Supplement: Supplementary file 1 [file 41390_2025_4499_MOESM1_ESM.pdf]

Table of contents:

Supplementary Methods: Study Definitions..2

Supplementary Figures...6

Supplementary Figure 1. Ethnicity 6

Supplementary Tables 7

Supplementary Table 1. Demographic features, SARS-CoV-2 exposure, and COVID vaccination status 7

Supplementary Table 2. MIS-C cohort. 8

Supplementary Table 3. MIS-C clinical phenotypes across MIS-C waves 13

Supplementary Table 4. MIS-C clinical phenotypes. 14

Appendix 20

Supplementary Methods: Study Definitions

The definition of Multisystem Inflammatory Syndrome in Children (MIS-C)included children who i) met the most permissive case definition according to international MIS-C and PIMS definitions1-4 and ii) were adjudicated in real-time by the physician at the bedside confirmed by the SickKids multidisciplinary MIS-C working group as having MIS-C and iii) were hospitalized and treated for the condition. The most permissive case definitions for MIS- C included the Royal College of Paediatrics and Child Health (RCPCH)[1], Canadian Paediatric Surveillance Program (CPSP)[2], World Health Organization (WHO)[3], and Centers for Disease Control and Prevention (CDC)[4] case definitions. Common elements to these definitions are the presence of prolonged fever, multiorgan dysfunction, laboratory evidence of hyperinflammation, with or without SARS-CoV-2 infection or exposure, and no alternative cause to explain the clinical presentation. RCPCH definition requires 1) persistent fever at > 38.5°C 2) Single organ or multiorgan dysfunction AND additional features (shock, cardiac, respiratory, renal, gastrointestinal or neurologic disorder), 3) neutrophilia, lymphopenia and elevated C-reactive protein (CRP). CPSP definition requires 1) fever > 38.0 °C for ≥ 3 days, 2) age < 18, 3) admission to hospital and features of Kawasaki disease (complete or incomplete) or toxic shock syndrome (typical or atypical), 4) elevated CRP, erythrocyte sedimentation rate (ESR) or ferritin. WHO definitions requires 1) ≥ 3 days of fever, 2) age between 0–19, 3) ≥ 2 features among admission to hospital and 2 of the following: rash, bilateral nonpurulent conjunctivitis, mucocutaneous inflammation signs (oral, hands or feet); hypotension or shock; cardiac involvement: features of myocardial dysfunction, pericarditis, valvulitis or coronary abnormalities (including echocardiography findings or elevated troponin/NT-proBNP); evidence of coagulopathy (by prothrombin time, partial thromboplastin time, elevated d-dimers); acute gastrointestinal problems (diarrhea, vomiting or abdominal pain), 4) elevated ESR, CRP or procalcitonin, 5) evidence of SARS-CoV-2 exposure. CDC definition requires 1) fever > 38.0 °C OR subjective ≥ 24 hours, 2) age < 21, 3) ≥ 2 organ systems involvement including cardiac, renal, respiratory, hematologic,

gastrointestinal, dermatologic or neurologic systems, 4) One or more of the following: elevated CRP, ESR, fibrinogen, procalcitonin, d-dimer, ferritin, LDH or IL-6; neutrophilia, lymphopenia, low albumin, 5) evidence of SARS-CoV-2 exposure.

Patients were considered to have been exposed to SARS-CoV-2 if 1) SARS-CoV-2 nucleic acid was detected by reverse transcription polymerase chain reaction (RT-PCR) on nasopharyngeal swab, 2) reactive IgG SARS-CoV-2 antibodies in serum (both nucleocapsid and spike when available), 3) patient had direct, close contact with a caregiver/household member who had a RT-PCR or Rapid Antigen Test (RAT) confirmed SARS-CoV-2 infection, or 4) patient had a positive SARS-CoV-2 RAT. Macrophage activation syndrome (MAS) was defined using the 2016 European League Against Rheumatism/American College of Rheumatology/Paediatric Rheumatology International Trials Organization classification criteria of MAS for systemic-onset juvenile idiopathic arthritis.[5]

MIS-C phenotypes included cardiogenic shock, Kawasaki disease (KD), and fever with hyperinflammation.[2,9]

Shock and KD shock syndrome were defined using previously established guidelines.[6-8] Complete and incomplete KD definitions were based on the American Heart Association (AHA) criteria.[8] Complete KD was defined with presence of ≥5 days of fever and the presence of ≥4 of the 5 principal clinical features including rash, peripheral extremity changes, oral mucosal changes, cervical lymphadenopathy (≥1.5 cm diameter), and bilateral bulbar conjunctival injection without exudate; and the diagnosis of incomplete KD, was based on presence of ≥5 days of fever with <4 clinical features with coronary artery lesions, or 2-3 clinical features of KD in the presence of supportive laboratory findings. Fever with hyperinflammation described patients who had fever and biochemical hyperinflammation but did not fulfill criteria for shock syndrome or KD.

Gastrointestinal abnormalities included any of the following: abdominal pain, vomiting, or diarrhea. Neurological features included headache or altered mental status. Renal abnormalities were defined as having at least one among proteinuria, hematuria, or acute kidney injury. Cardiac abnormalities were defined as having at least one of the following: i) coronary artery abnormality defined as body

surface area normalized Z-score >2; ii) electrocardiogram (ECG) abnormality defined as prolonged PR interval, T wave inversion, ST-segment elevation or depression; iii) ventricular dysfunction defined as ejection fraction <55% on echocardiogram; iv) elevated troponin; and/or v) elevated and N-terminal pro-brain natriuretic peptide.

Severity measures included length of hospital stay, paediatric intensive care unit admission, respiratory support, fluid resuscitation and inotropic support. Complications included shock, MAS and arthritis. Cardiac outcomes included i) coronary artery abnormality defined as body surface area normalized Z-score >2, defined as severe coronary artery aneurysms if Z-score >5; ii) ECG abnormality defined as prolonged PR interval, T wave inversion, ST-segment elevation or depression which have been calculated over a mean follow-up period of 49.08 days.

1 Royal College of Paediatrics and Child Health. Guidance: paediatric multisystem inflammatory syndrome temporally associated with COVID-19; 2020. Available from:https://www.rcpch.ac.uk/resources/paediatric-multisystem-inflammatory-syndrome-temporally-associated-covid-19-pims-guidance (accessed December 13, 2023).

2 Berard, RA. et al. Acute Care Committee. Paediatric inflammatory multisystem syndrome temporally associated with COVID-19. Canadian Paediatric Society Jul 6, 2020. Available from: https://www.cps.ca/en/documents/position/pims (accessed December 13, 2023).

3 World Health Organization. Multisystem inflammatory syndrome in children and adolescents with COVID-19. Scientific brief, 15 May 2020. Geneva, Switzerland: WHO; 2020. Available from: https://apps.who.int/iris/handle/10665/332095 (accessed December 13, 2023).

4 US Centers for Disease Control and Prevention, Emergency Preparedness and Response. Multisystem Inflammatory Syndrome in Children (MIS-C) Associated with Coronavirus Disease 2019 (COVID-19). May 14, 2020. Available from: https://emergency.cdc.gov/han/2020/han00432.asp (accessed December 13, 2023).

5 Ravelli, A. et al. 2016 Classification Criteria for Macrophage Activation Syndrome Complicating Systemic Juvenile Idiopathic Arthritis: A European League Against Rheumatism/American College of Rheumatology/Paediatric Rheumatology International Trials Organisation Collaborative Initiative. *Arthritis Rheumatol.* **68**(3): 566-76 (2016).

6 Ralston, M. et al. *Pediatric Advanced Life Support Provider Manual* (American Heart Association , Dallas, TX, 2006).

7 Fitzmaurice, L., Gerardi, M.J. Cardiovascular system. In *APLS: The Pediatric Emergency Medicine Resource*. 4th ed (Gausche-Hill, M. Fuchs, S.,Yamamoto, L, eds.) 106-145 (Jones and Bartlett; Sudbury,MA, 2007).

8 McCrindle, BW. et al. Diagnosis, Treatment, and Long-Term Management of Kawasaki Disease: A Scientific Statement for Health Professionals From the American Heart Association. *Circulation.* **135**(17): e927-e999 (2017).

9 Cherqaoui, B. et al. Delineating phenotypes of Kawasaki disease and SARS-CoV-2-related inflammatory multisystem syndrome: a French study and literature review. *Rheumatology* (Oxford). **60**(10): 4530-4537 (2021).

Supplementary Figures

Supplementary Figure S1. Ethnicity

Self-reported ethnicity for A) Population of the city of Toronto, B) Confirmed COVID-19 cases in Toronto in 2020, C) Pre-pandemic KD (2019), D) Ancestral/pre-Delta MIS-C cohort, E) Delta MIS-C cohort, F) Omicron MIS-C cohort

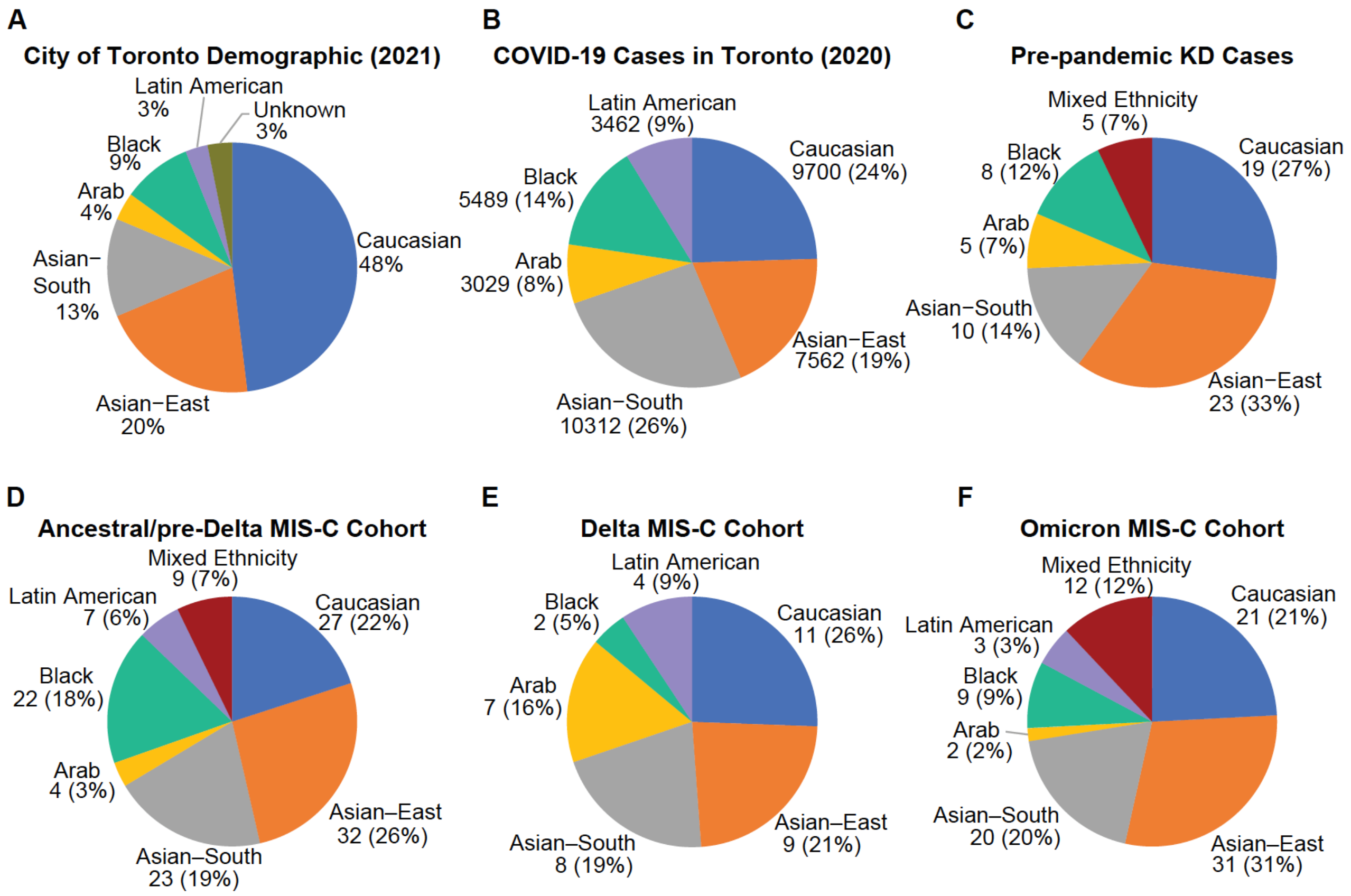

Supplementary Tables

Supplementary Table S1. Demographic features, SARS-CoV-2 exposure, and COVID vaccination status

	**n = 384**[a]
**Age at diagnosis, years**	5.0 (2.0, 9.0)
**Sex**	
F	162 (42%)
M	222 (58%)
**BMI**	16.5 (15.2, 19.0)
**Confirmed SARS-CoV-2 exposure**	178 (46%)
**COVID vaccine: dose 1**	34 / 334 (10%)
**COVID vaccine: dose 2**	26 / 333 (8%)
**COVID vaccine: dose 3**	6 / 329 (2%)

Abbreviation: BMI, Body Mass Index; COVID-19, coronavirus disease 2019.

[a]Median (IQR); No. (%); n / N (%)

Supplementary Table S2. MIS-C cohort. Demographic features, clinical and laboratory findings at presentation, severity[c], complications[d], and cardiac outcomes[e] according to MIS-C waves

	**Ancestral/pre-Delta**, (n = 140[a])	**Delta**, (n = 71[a])	**Omicron**, (n = 173[a])	**p-value**[b]
**Age at diagnosis, years**	4.5 (2.0, 9.0)	5.0 (2.5, 10.0)	5.0 (3.0, 8.0)	0.9
**Sex of child**				>0.9
F	60 (43%)	29 (41%)	73 (42%)	
M	80 (57%)	42 (59%)	100 (58%)	
**BMI**	16.8 (15.2, 19.4)	17.3 (15.7, 20.1)	16.2 (14.9, 17.6)	0.028*
**Confirmed SARS-CoV-2 exposure**	49 (35%)	39 (55%)	90 (52%)	0.003*
**Features at presentation**				
**Duration of fever, days**	5.0 (4.0, 7.0)	5.0 (5.0, 6.5)	6.0 (5.0, 7.0)	0.039
**Gastrointestinal features**	108 (77%)	56 (79%)	107 (62%)	0.003*
**Neurologic features**	45 (32%)	27 (38%)	53 (31%)	0.5
**Renal abnormalities**	93 (66%)	31 (44%)	85 (49%)	0.001*
**Coagulation abnormalities**	121 / 122 (99%)	57 / 57 (100%)	101 / 101 (100%)	>0.9

	**Ancestral/pre-Delta**, (n = 140[a])	**Delta**, (n = 71[a])	**Omicron**, (n = 173[a])	**p-value**[b]
**Cardiac abnormalities**	134 (96%)	64 (90%)	132 (76%)	<0.001*
**White blood cell (4.0-13.8 x 10^9/L)**	11.8 (7.4, 15.6)	10.3 (6.8, 14.9)	12.5 (8.0, 16.5)	0.14
**Absolute Neutrophil Count (1.6-8.3 x 10^9/L)**	6.5 (3.4, 9.5)	5.8 (2.8, 9.6)	7.4 (4.5, 11.1)	0.022*
**Absolute Lymphocyte Count (1.3-5.8 x 10^9/L)**	1.8 (1.0, 3.7)	1.6 (0.9, 2.5)	1.9 (1.0, 3.3)	0.2
**Hemoglobin (102-127 g/L)**	109.0 (101.8, 119.0)	108.0 (98.0, 118.0)	110.0 (102.0, 118.5)	0.6
**Platelet count (189-394 x 10^9/L)**	211.0 (141.0, 364.0)	227.0 (147.0, 342.0)	302.0 (192.0, 413.0)	0.002*
**C-reactive protein (0.1-1 mg/L)**	133.9 (78.5, 190.1)	134.9 (82.1, 214.0)	113.7 (56.4, 168.3)	0.030*
**Ferritin (5.3-99.9 ug/L)**	280.0 (156.9, 638.5)	465.0 (214.6, 756.1)	270.2 (157.4, 475.0)	0.041*
**Sodium (135-143 mmol/L)**	136.0 (134.0, 138.0)	137.0 (133.8, 138.3)	135.0 (133.0, 137.0)	0.038*
**Albumin (35-47 g/L)**	36.0 (32.0, 40.0)	33.0 (29.0, 36.0)	35.0 (31.0, 39.0)	0.006*
**Creatinine (16-35 umol/L)**	27.5 (20.8, 44.3)	32.0 (24.0, 50.0)	27.0 (21.0, 46.0)	0.14
**Alanine aminotransferase (<25 U/L)**	26.0 (16.0, 49.0)	31.0 (21.0, 57.5)	32.0 (19.0, 87.0)	0.011*
**Aspartate aminotransferase (<52 U/L)**	51.0 (35.0, 87.8)	47.0 (30.0, 65.0)	49.5 (32.0, 85.8)	0.3
**Absolute Band (0-0.01 x 10^9/L)**	1.4 (0.6, 3.2)	2.3 (1.5, 4.7)	2.2 (1.1, 3.7)	0.10

	**Ancestral/pre-Delta**, (n = 140[a])	**Delta**, (n = 71[a])	**Omicron**, (n = 173[a])	**p-value**[b]
**Erythrocyte sedimentation rate (mm/h)**	55.0 (31.0, 83.0)	59.5 (33.3, 74.8)	63.0 (46.3, 88.0)	0.064
**Lactate Dehydrogenase (500-920 IU/L)**	782.0 (627.0, 1,084.5)	745.0 (611.0, 955.0)	783.0 (525.0, 1,007.0)	0.2
**Triglycerides (<0.85 mmol/L)**	1.8 (1.4, 2.5)	1.7 (1.4, 2.4)	1.8 (1.4, 2.4)	0.8
**INR (0.8-1.2)**	1.2 (1.1, 1.4)	1.2 (1.1, 1.4)	1.2 (1.1, 1.3)	0.8
**PTT (24-36 s)**	31.0 (28.0, 34.0)	32.3 (29.3, 34.8)	33.1 (29.4, 36.0)	0.034*
**Fibrinogen (1.9-4.3 g/L)**	5.2 (4.1, 6.4)	5.6 (4.7, 6.8)	5.3 (4.3, 6.8)	0.4
**D-dimer (<0.5 ug/mL)**	1.6 (1.0, 3.1)	2.4 (1.6, 3.7)	2.0 (1.2, 4.3)	0.011*
**Troponin (<10 ng/L)**	10.0 (10.0, 41.5)	10.6 (10.0, 75.8)	10.0 (10.0, 31.5)	0.094
**NT-proBNP (<125 ng/L)**	667.3 (187.6, 2,403.2)	2,013.3 (521.0, 7,135.0)	681.7 (202.4, 3,277.4)	0.014*
**Severity measures**				
**Length of hospital stay, days**	4.0 (3.0, 6.0)	4.0 (3.5, 6.0)	4.0 (3.0, 5.0)	0.010*
**PICU admission**	26 / 140 (19%)	22 / 71 (31%)	27 / 171 (16%)	0.024*
**Respiratory support**	15 / 140 (11%)	9 / 71 (13%)	11 / 171 (6%)	0.2
**Fluid resuscitation**	0 (0%)	13 (18%)	29 (17%)	<0.001*

	**Ancestral/pre-Delta**, (n = 140[a])	**Delta**, (n = 71[a])	**Omicron**, (n = 173[a])	**p-value**[b]
**Inotropic support**	20 / 140 (14%)	15 / 71 (21%)	24 / 171 (14%)	0.3
**Complications**				
**Shock**	22 / 140 (16%)	25 / 71 (35%)	29 / 171 (17%)	0.002*
**Macrophage Activation Syndrome**	17 / 140 (12%)	14 / 71 (20%)	21 / 171 (12%)	0.3
**Arthritis**	5 / 140 (4%)	1 / 71 (1%)	10 / 171 (6%)	0.3
**Cardiac outcomes**				
**Coronary artery lesion**	29 / 139 (21%)	15 / 69 (22%)	32 / 153 (21%)	>0.9
**Abnormal ECG**	35 / 139 (25%)	21 / 71 (30%)	33 / 168 (20%)	0.2
**Z-score maximum**	1.5 (1.0, 1.9)	1.6 (1.1, 1.9)	1.4 (1.0, 1.9)	0.6
**Severe coronary aneurysms**	4 / 139 (3%)	1 / 69 (1%)	8 / 153 (5%)	0.4

Abbreviation: BMI, Body Mass Index; NT-proBNP, N-terminal pro-brain natriuretic peptide; PICU, paediatric intensive care unit; ECG, electrocardiogram.

SI conversion factors: To convert ALT and lactate dehydrogenase to µkat/L, multiply by 0.0167; to convert creatinine to mg/dL, divide by 88.4. To convert D-dimer to nmol/L multiply by 5.476.

[a]Median (IQR); No. (%); n / N (%)

[b]Kruskal-Wallis rank sum test; Pearson's Chi-squared test; Fisher's exact test

*p value <0.05

[c] Severity measures: Length of hospital stay, PICU admission, Respiratory support, Fluid resuscitation, Inotropic support

[d] Complication: Shock, MAS, arthritis

[e] Cardiac outcomes:

i. coronary artery abnormality defined as body surface area normalized Z-score >2;
ii. ECG abnormality defined as prolonged PR interval, T wave inversion, ST-segment elevation or depression
iii. Severe coronary aneurysms: Z-score >5

Supplementary Table S3. MIS-C clinical phenotypes across MIS-C waves A) for children satisfying the WHO MIS-C case definition B) for children with confirmed SARS-CoV-2 Exposure

A)

MIS-C Phenotype	**Ancestral/pre-Delta**, (n= 43[a])	**Delta**, (n = 37[a])	**Omicron**, (n = 84[a])
**Fever and hyperinflammation**	11 (26%)	5 (14%)	4 (5%)
**KD**	19 (44%)	12 (32%)	62 (74%)
**Shock**	13 (30%)	20 (54%)	18 (21%)

B)

MIS-C Phenotype	**Ancestral/pre-Delta**, (n = 49[a])	**Delta**, (n = 39[a])	**Omicron**, (n = 90[a])
**Fever and hyperinflammation**	12 (24%)	6 (15%)	4 (4%)
**KD**	24 (49%)	12 (31%)	67 (74%)
**Shock**	13 (27%)	21 (54%)	19 (21%)

Abbreviation: KD, Kawasaki disease.

[a]No. (%)

Supplementary Table S4. MIS-C clinical phenotypes. Demographic, clinical and laboratory findings at presentation, severity[c], complications[d], and cardiac outcomes[e] according to MIS-C clinical phenotypes

	**Shock**, (n = 76[a])	**KD**, (n = 264[a])	**Fever with hyperinflammation**, (n = 44[1])	**p-value**[b]
**Age at diagnosis, years**	9.0 (5.0, 13.0)	4.0 (2.0, 7.0)	3.0 (1.0, 8.3)	<0.001*
**Sex of child**				0.3
F	35 (46%)	105 (40%)	22 (50%)	
M	41 (54%)	159 (60%)	22 (50%)	
**BMI**	17.0 (15.5, 22.8)	16.4 (15.2, 18.5)	16.1 (15.6, 18.1)	0.3
**Confirmed SARS-CoV-2 exposure**	53 (70%)	103 (39%)	22 (50%)	<0.001*
**Features at presentation**				
**Duration of fever, days**	5.0 (4.0, 6.0)	6.0 (5.0, 7.0)	5.0 (4.0, 7.0)	<0.001*
**Number of KD features**	3.0 (1.0, 4.0)	3.0 (3.0, 4.0)	0.5 (0.0, 1.0)	<0.001*
**Rash**	39 (51%)	207 (78%)	8 (18%)	<0.001*
**Peripheral extremity changes**	23 (30%)	167 (63%)	0 (0%)	<0.001*
**Cervical lymphadenopathy**	27 (36%)	107 (41%)	2 (5%)	<0.001*

	**Shock**, (n = 76[a])	**KD**, (n = 264[a])	**Fever with hyperinflammation**, (n = 44[1])	**p-value**[b]
**Oromucosal changes**	40 / 76 (53%)	204 / 263 (78%)	3 / 44 (7%)	<0.001*
**Bilateral non-exudative conjunctivitis**	64 (84%)	205 (78%)	9 (20%)	<0.001*
**Complete KD**	21 (28%)	130 (49%)	0 (0%)	<0.001*
**Gastrointestinal features**	68 (89%)	168 (64%)	35 (80%)	<0.001*
**Neurologic features**	37 (49%)	73 (28%)	15 (34%)	0.003*
**Renal abnormalities**	54 (71%)	126 (48%)	29 (66%)	<0.001*
**Coagulation abnormalities**	70 / 70 (100%)	174 / 175 (99%)	35 / 35 (100%)	>0.9
**Cardiac abnormalities**	74 (97%)	218 (83%)	38 (86%)	0.005*
**White blood cell (4.0-13.8 x 10^9/L)**	10.9 (7.6, 14.8)	12.4 (7.7, 16.4)	10.2 (7.2, 15.8)	0.2
**Absolute Neutrophil Count (1.6-8.3 x 10^9/L)**	7.0 (3.6, 10.5)	7.2 (4.0, 10.5)	5.7 (2.8, 9.2)	0.3
**Absolute Lymphocyte Count (1.3-5.8 x 10^9/L)**	1.0 (0.5, 1.5)	2.4 (1.2, 3.7)	1.9 (1.0, 3.8)	<0.001*
**Hemoglobin (102-127 g/L)**	108.0 (99.0, 116.5)	110.0 (102.0, 119.0)	108.0 (99.0, 118.5)	0.5
**Platelet count (189-394 x 10^9/L)**	145.0 (103.0, 219.3)	310.0 (202.8, 415.8)	200.0 (153.0, 356.0)	<0.001*

	**Shock**, (n = 76[a])	**KD**, (n = 264[a])	**Fever with hyperinflammation**, (n = 44[1])	**p-value**[b]
**C-reactive protein (0.1-1 mg/L)**	171.8 (116.4, 241.6)	100.4 (54.2, 158.5)	141.8 (89.0, 209.5)	<0.001*
**Ferritin (5.3-99.9 ug/L)**	640.8 (390.8, 1,707.7)	218.9 (138.3, 441.7)	268.3 (194.9, 542.6)	<0.001*
**Sodium (135-143 mmol/L)**	134.0 (131.0, 137.0)	136.0 (134.0, 138.0)	137.0 (134.8, 138.0)	<0.001*
**Albumin (35-47 g/L)**	29.0 (25.0, 33.0)	36.0 (33.0, 39.0)	37.0 (33.5, 40.5)	<0.001*
**Creatinine (16-35 umol/L)**	52.5 (34.0, 83.0)	26.0 (20.0, 37.0)	26.0 (19.0, 35.5)	<0.001*
**Alanine aminotransferase (<25 U/L)**	36.0 (23.0, 67.0)	31.0 (19.0, 74.8)	18.0 (14.0, 31.5)	<0.001*
**Aspartate aminotransferase (<52 U/L)**	63.0 (37.5, 97.0)	45.5 (32.0, 77.0)	47.0 (33.8, 68.3)	0.2
**Absolute Band (0-0.01 x 10^9/L)**	2.8 (1.5, 5.1)	1.7 (0.8, 2.9)	2.1 (0.6, 3.9)	0.006*
**Erythrocyte sedimentation rate (mm/h)**	56.5 (23.0, 81.5)	60.0 (38.5, 84.5)	66.0 (36.3, 85.5)	0.2
**Lactate Dehydrogenase (500-920 IU/L)**	774.0 (585.0, 1,157.0)	787.5 (607.0, 1,007.5)	715.0 (586.0, 995.0)	0.9
**Triglycerides (<0.85 mmol/L)**	2.0 (1.6, 3.0)	1.7 (1.3, 2.3)	1.8 (1.5, 2.5)	0.029*
**INR (0.8-1.2)**	1.3 (1.1, 1.5)	1.2 (1.1, 1.3)	1.1 (1.0, 1.4)	0.073
**PTT (24-36 s)**	33.1 (29.2, 35.0)	32.0 (28.4, 35.1)	32.0 (30.0, 34.7)	0.7

	**Shock**, (n = 76[a])	**KD**, (n = 264[a])	**Fever with hyperinflammation**, (n = 44[1])	**p-value**[b]
**Fibrinogen (1.9-4.3 g/L)**	5.2 (4.0, 7.0)	5.3 (4.3, 6.5)	6.0 (4.5, 7.1)	0.4
**D-dimer (<0.5 ug/mL)**	2.6 (1.8, 5.2)	1.6 (1.0, 3.5)	1.8 (1.0, 2.3)	<0.001*
**Troponin (<10 ng/L)**	73.2 (10.2, 340.0)	10.0 (10.0, 10.0)	10.0 (10.0, 16.1)	<0.001*
**NT-proBNP (<125 ng/L)**	3,614.2 (1,667.5, 13,470.3)	526.0 (183.2, 1,784.1)	377.1 (172.6, 1,959.1)	<0.001*
**Severity measures**				
**Length of hospital stay, days**	6.0 (4.0, 9.5)	4.0 (3.0, 5.0)	4.0 (3.0, 5.3)	<0.001*
**PICU admission**	63 / 75 (84%)	9 / 263 (3%)	3 / 44 (7%)	<0.001*
**Respiratory support**	25 / 75 (33%)	6 / 263 (2%)	4 / 44 (9%)	<0.001*
**Fluid resuscitation**	20 (26%)	19 (7%)	3 (7%)	<0.001*
**Inotropic support**	54 / 75 (72%)	3 / 263 (1%)	2 / 44 (5%)	<0.001*
**Complications**				
**Shock**	76 / 76 (100%)	0 / 262 (0%)	0 / 44 (0%)	<0.001*
**Macrophage Activation Syndrome**	25 / 76 (33%)	25 / 262 (10%)	2 / 44 (5%)	<0.001*

	**Shock**, (n = 76[a])	**KD**, (n = 264[a])	**Fever with hyperinflammation**, (n = 44[1])	**p-value**[b]
**Arthritis**	1 / 76 (1%)	14 / 262 (5%)	1 / 44 (2%)	0.3
**Cardiac outcomes**				
**Coronary artery lesion**	19 / 73 (26%)	57 / 246 (23%)	0 / 42 (0%)	0.002*
**Abnormal ECG**	36 / 75 (48%)	46 / 259 (18%)	7 / 44 (16%)	<0.001*
**Z-score maximum**	1.6 (1.2, 2.1)	1.5 (1.0, 1.9)	1.3 (0.8, 1.5)	0.032*
**Severe coronary aneurysms**	2 / 73 (3%)	11 / 246 (5%)	0 / 42 (0%)	0.5

Abbreviation: BMI, Body Mass Index; NT-proBNP, N-terminal pro-brain natriuretic peptide; PICU, paediatric intensive care unit; ECG, electrocardiogram.

SI conversion factors: To convert ALT and lactate dehydrogenase to µkat/L, multiply by 0.0167; to convert creatinine to mg/dL, divide by 88.4. To convert D-dimer to nmol/L multiply by 5.476.

[1]Median (IQR); No. (%); n / N (%)

[2]Kruskal-Wallis rank sum test; Pearson's Chi-squared test; Fisher's exact test

*p value <0.05

[c] Severity measures: Length of hospital stay, PICU admission, Respiratory support, Fluid resuscitation, Inotropic support

[d] Complication: Shock, MAS, arthritis

[e] Cardiac outcomes:

i. coronary artery abnormality defined as body surface area normalized Z-score >2;
ii. ECG abnormality defined as prolonged PR interval, T wave inversion, ST-segment elevation or depression
iii. Severe coronary aneurysms: Z-score >5

Appendix

Consortia authorship list: SickKids MIS-C Working Group (alphabetical order):

- Nita Chahal NPPeds PhD
  - Division of Cardiology, Department of Paediatrics, The Hospital for Sick Children, University of Toronto, Toronto, Canada
- Dilan Dissanayake MD PhD
  - Division of Rheumatology, Department of Paediatrics, The Hospital for Sick Children, University of Toronto, Toronto, Canada
  - Cell and Systems Biology Program, The Hospital for Sick Children Research Institute, Toronto, Canada
- Tala El Tal MD MSc
  - Division of Rheumatology and Dermatology, Department of Paediatrics, Children's Hospital of Eastern Ontario, Ottawa, Canada
- Jeremy N Friedman MB.ChB
  - Department of Paediatrics, The Hospital for Sick Children, University of Toronto, Toronto, Canada
- Beth D. Gamulka MDCM
  - Department of Paediatrics, The Hospital for Sick Children, University of Toronto, Toronto, Canada
- Ellen Go MD
  - Division of Rheumatology, Department of Paediatrics, The Hospital for Sick Children, University of Toronto, Toronto, Canada
  - Cell and Systems Biology Program, The Hospital for Sick Children Research Institute, Toronto, Canada
  - Division of Pediatric Rheumatology, Indiana University Health Medical Center, Atlanta, GA, USA
- Charlotte Moore Hepburn MD
  - Department of Paediatrics, The Hospital for Sick Children, University of Toronto, Toronto, Canada
- Linda T. Hiraki MD, ScD
  - Division of Rheumatology, Department of Paediatrics, The Hospital for Sick Children, University of Toronto, Toronto, Canada
- Julie Johnstone MD

    - Department of Paediatrics, The Hospital for Sick Children, University of Toronto, Toronto, Canada
- Andrea Knight MD
    - Division of Rheumatology, Department of Paediatrics, The Hospital for Sick Children, University of Toronto, Toronto, Canada
- Raphaël Kraus MD
    - Division of Rheumatology, Department of Paediatrics, Centre Hospitalier Universitaire Sainte-Justine, Montreal, Quebec, Canada
    - The Rosalind & Morris Goodman Family Pediatric Formulations Centre of the CHU Sainte-Justine, Montreal, Quebec, Canada
- Ronald M Laxer MD
    - Division of Rheumatology, Departments of Paediatrics, The Hospital for Sick Children, University of Toronto, Toronto, Canada
    - Division of Rheumatology, St. Michael's Hospital, Toronto, Canada
- Deborah M Levy MD
    - Division of Rheumatology, Department of Paediatrics, The Hospital for Sick Children, University of Toronto, Toronto, Canada
- Jayne M MacMahon MB BCH BAO
    - Division of Rheumatology, Department of Paediatrics, The Hospital for Sick Children, University of Toronto, Toronto, Canada
- Brian W McCrindle MD MSc
    - Division of Cardiology, Department of Paediatrics, The Hospital for Sick Children, University of Toronto, Toronto, Canada
- Luc Mertens MD
    - Division of Cardiology, Department of Paediatrics, The Hospital for Sick Children, University of Toronto, Toronto, Canada
- Trent Mizzi MD
    - Division of Emergency Medicine, Department of Paediatrics, The Hospital for Sick Children, University of Toronto, Toronto, Canada
- Shaun K Morris MD
    - Division of Infectious Disease, Department of Paediatrics, The Hospital for Sick Children, University of Toronto, Toronto, Canada
- Laila Premji MD

  - Department of Paediatrics, The Hospital for Sick Children, University of Toronto, Toronto, Canada
- Lynn Spiegel MD
  - Division of Rheumatology, Department of Paediatrics, The Hospital for Sick Children, University of Toronto, Toronto, Canada
- Shirley ML Tse MD
  - Division of Rheumatology, Department of Paediatrics, The Hospital for Sick Children, University of Toronto, Toronto, Canada
- Paul Tsoukas MD
  - Division of Rheumatology, Department of Paediatrics, The Hospital for Sick Children, University of Toronto, Toronto, Canada
  - Cell and Systems Biology Program, The Hospital for Sick Children Research Institute, Toronto, Canada
- Rae S M Yeung MD PhD
  - Division of Rheumatology, Department of Paediatrics, The Hospital for Sick Children, University of Toronto, Toronto, Canada
  - Cell and Systems Biology Program, The Hospital for Sick Children Research Institute, Toronto, Canada
  - Department of Immunology and Institute of Medical Science, University of Toronto, Canada
